# Melatonin Mitigates Acidosis-Induced Neuronal Damage by Up-Regulating Autophagy via the Transcription Factor EB

**DOI:** 10.3390/ijms26031170

**Published:** 2025-01-29

**Authors:** Yan Shi, Zhaoyu Mi, Wei Zhao, Yue Hu, Hui Xiang, Yaoxue Gan, Shishan Yuan

**Affiliations:** 1 Key Laboratory of Study and Discovery of Small Targeted Molecules of Hunan Province, School of Pharamceutical Sciences, Health Science Center, Hunan Normal University, Changsha 410013, China; shiy@hunnu.edu.cn; 2 School of Medical Technology and Translational Medicine, Hunan Normal University, Changsha 410006, China; zoeym0323@hunnu.edu.cn (Z.M.); zzzzzw@hunnu.edu.cn (W.Z.); 202220193561@hunnu.edu.cn (Y.H.); 202320193536@hunnu.edu.cn (H.X.); kwx0130@hunnu.edu.cn (Y.G.); 3 Engineering Research Center of Reproduction and Translational Medicine of Hunan Province, Health Science Center, Hunan Normal University, Changsha 410013, China

**Keywords:** TFEB, acidosis, melatonin, autophagy, neuroprotective

## Abstract

Acidosis, a common feature of cerebral ischemia and hypoxia, results in neuronal damage and death. This study aimed to investigate the protective effects and mechanisms of action of melatonin against acidosis-induced neuronal damage. SH-SY5Y cells were exposed to an acidic environment to simulate acidosis, and a photothrombotic (PT) infarction model was used to establish an animal model of cerebral ischemia of male C57/BL6J mice. Both in vivo and in vitro studies demonstrated that acidosis increased cytoplasmic transcription factor EB (TFEB) levels, reduced nuclear TFEB levels, and suppressed autophagy, as evidenced by elevated p62 levels, a higher LC3-II/LC3-I ratio, decreased synapse-associated proteins (PSD-95 and synaptophysin), and increased neuronal apoptosis. In contrast, melatonin promoted the nuclear translocation of TFEB, enhanced autophagy, and reversed neuronal apoptosis. Moreover, the role of TFEB in melatonin’s neuroprotective effects was validated by modulating TFEB nuclear translocation. In conclusion, melatonin mitigates acidosis-induced neuronal damage by promoting the nuclear translocation of TFEB, thereby enhancing autophagy. These findings offer new insights into potential treatments for acidosis.

## 1. Introduction

The brain is at the center of the body and regulates metabolism and movement. Acid-base imbalances, especially acidosis, occur when the supply, production, utilization, or storage of energy in the brain are inadequate. Acidosis, a pathological condition, is characterized by significant pathophysiological alterations following ischemia and hypoxia [1]. Under normal physiological conditions, the extracellular pH of brain tissue fluctuates between 7.2 and 7.4, while in ischemic brain tissue, the pH can drop to 6.5–6.0 [2,3]. In a rodent model of acute middle cerebral artery occlusion (MCAO), the tissue pH in the ischemic core may decrease to 6.0 [4]. Acidosis is a common feature of most neurological diseases, which can lead to neuronal damage and death [1]. Reduced cerebral perfusion is observed in Alzheimer’s disease (AD), which leads to a hypoxic and acidic microenvironment [5]. Acidosis increases amyloid β-protein (Aβ) expression in hippocampal neurons, promoting Aβ accumulation [6]. Lactic acidosis is observed in patients with Parkinson’s disease (PD) and PD mouse models [7,8]. Since maintaining pH stability is crucial for normal brain function, elucidating the mechanisms of acidosis-induced neuronal damage and exploring protective strategies are critical for brain protection.

Autophagy is an essential adaptive response for maintaining cellular homeostasis during pathological conditions such as ischemia and hypoxia-induced injury [9]. This self-digestive process preserves cellular homeostasis by degrading and recycling damaged organelles and misfolded protein aggregates [10,11]. Mammalian autophagy includes chaperone-mediated autophagy, microautophagy, and macroautophagy (commonly referred to as autophagy). Macroautophagy is highly associated with neurodegenerative diseases [12]. Under normal conditions, neurons efficiently degrade autophagosomes through lysosomes. However, impaired autophagosome clearance leads to their accumulation, adversely affecting neuronal health [13]. An elevated intralysosomal pH in ischemic tissues following ischemic stroke hinders lysosome–autophagosome fusion, causing substrate degradation failure. This can also result in the formation of abnormal acidified autolysosomes, reducing degradation efficiency [14]. As ischemia progresses, lysosome–autophagosome fusion is further impaired, leading to abnormal autophagic substrate accumulation and disrupted autophagic flux, ultimately damaging the neurons [15]. Ensuring the smooth progression of autophagy can protect neurons from damage.

Transcription factor EB (TFEB) is pivotal in regulating autophagy and lysosome biogenesis. It controls autophagosome formation, facilitates lysosome fusion, and ensures lysosomal function [16,17]. Phosphorylated TFEB remains in the cytoplasm, whereas dephosphorylated TFEB is translocated to the nucleus, where it promotes the transcriptional upregulation of lysosome/autophagy genes such as *ATG5* and *ATG7* by directly binding to the Coordinated Lysosomal Expression and Regulation (CLEAR) elements in their promoters [18,19,20]. Relevant studies have shown that TFEB activation enhances autophagy, promoting the degradation of abnormal proteins such as Aβ and tau [21]. Activating TFEB in AD mouse models improves lysosomal biogenesis, alleviates lysosomal clearance defects, increases Aβ degradation efficiency, reduces neurotoxicity, and enhances memory [22]. Targeted TFEB therapy has shown potential in addressing autophagy dysfunction in neurological diseases [23].

Melatonin, chemically known as n-acetyl-5-methoxy tryptophan, is a tryptophan derivative primarily synthesized by the pineal gland at night. It exhibits diverse biological effects, including antioxidant, anti-inflammatory, neuroprotective, and anti-injury properties [24]. Moreover, it stabilizes synaptic structure, enhances synaptic function, and promotes cognitive function [25]. Dysregulated melatonin expression and its secretory rhythm are closely related to aging and neurodegenerative diseases. Due to its various biological effects, melatonin protects against a range of nervous system diseases [26,27]. Moreover, melatonin protects against the neuronal death, synaptic protein reduction, and tau protein hyperphosphorylation induced by acidic environments [28]. It also reverses abnormal autophagy–lysosome signaling in neurons exposed to acidic conditions [28,29]. Although melatonin has been extensively studied in neurodegenerative diseases such as AD and PD, the effect and mechanism of melatonin on acidosis associated with neurological diseases have not been reported yet [26,30,31]. Further studies are required to determine whether melatonin exerts similar effects in vivo. Recent studies have focused on melatonin’s antioxidant and anti-inflammatory properties; however, the specific mechanisms underlying these effects remain unclear.

Our previous study showed that melatonin protects neurons in acidic environments [28]. However, the specific roles of TFEB and autophagy in these processes require further investigation to assess their potential as therapeutic targets. In this study, we showed that melatonin promotes TFEB nuclear translocation in a cerebral ischemia model, enhancing autophagy and reducing neuronal death. Specifically, TFEB nuclear translocation decreased in the cortex of mice with PT-stroke and SH-SY5Y cells exposed to acidic environments. Melatonin reversed these effects by promoting TFEB nuclear translocation, enhancing autophagy, improving synaptic function, and reducing neuronal apoptosis. Our findings suggest that TFEB activation through melatonin may be a promising therapeutic strategy for treating acidosis by enhancing autophagy and may provide new therapeutic targets for neurological diseases associated with acidosis.

## 2. Results

### 2.1. Acidosis Inhibited TFEB Nuclear Translocation and Autophagy in Neurons

**Figure 1:**
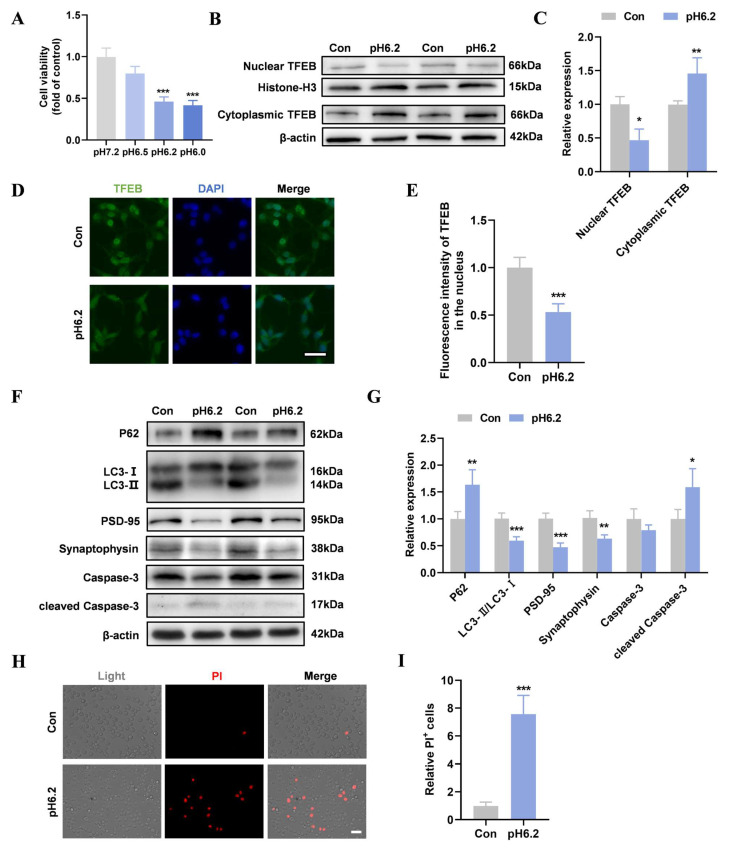
Acidosis inhibited TFEB nuclear translocation and autophagy in neurons. (**A**) SH-SY5Y cells were treated with different pH values (pH 6.5, pH 6.2, and pH 6.0) for 24 h and CCK-8 was performed to detect cell viability. *** *p* < 0.001 vs. pH 7.2, N = 3/group. (**B**,**C**) The Western blotting of cytoplasmic and nuclear TFEB and quantitative analysis. (**D**,**E**) Immunofluorescence was performed to detect the distribution of TFEB in SH-SY5Y cells. TFEB signals in the nucleus were quantified. (Scale bar = 20 µm). (**F**,**G**) The Western blotting of P62, LC3, PSD-95, synaptophysin, Caspase-3, and cleaved Caspase-3 and quantitative analysis. (**H**,**I**) PI staining was performed to validate and quantify the cell death ratio (Scale bar = 20 µm). Data are presented as means ± SD. * *p* < 0.05, ** *p* < 0.01, *** *p* < 0.001 vs. Con, N = 4/group.

SH-SY5Y cell viability decreased after 24 h in media with different pH values (6.5, 6.2, and 6.0). There was a significant decrease from the control group (pH 7.2) in cell viability in media with pH 6.2 and pH 6.0 (Figure 1A). Therefore, pH 6.2 was selected for long-term culture. Western blotting and immunofluorescence analysis showed that acidosis increased cytoplasmic TFEB levels and decreased nuclear TFEB levels in SH-SY5Y cells (Figure 1B–E). In the pH 6.2 group, p62 levels increased, and LC3-II/LC3-I levels decreased (Figure 1F,G), indicating significant inhibition of autophagy in SH-SY5Y cells in an acidic environment. Furthermore, synaptic damage and increased apoptosis were observed under acidic conditions, as evidenced by decreased levels of PSD-95 and synaptophysin and increased cleaved Caspase-3 levels (Figure 1F,G). Additionally, the PI staining showed a significantly higher proportion of PI-positive cells in the pH 6.2 group compared to the control group (Figure 1H,I).

### 2.2. Melatonin Restored TFEB Nuclear Translocation and Autophagy in Neurons

**Figure 2:**
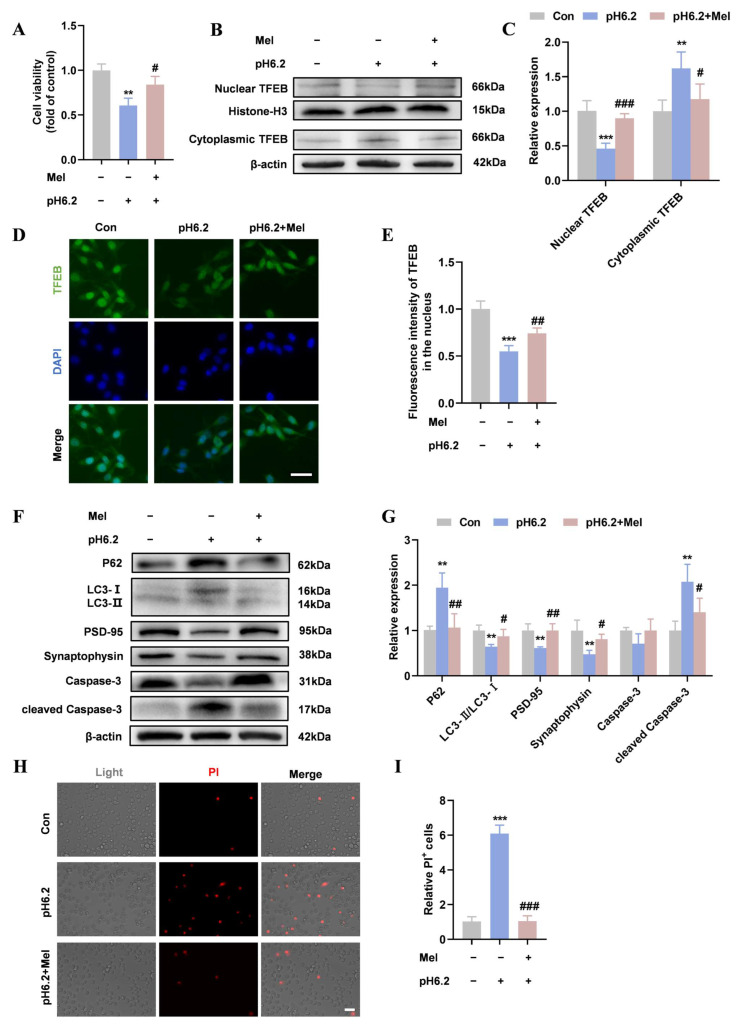
Melatonin restored TFEB nuclear translocation and autophagy in neurons. (**A**) SH-SY5Y cells were treated with pH 6.2 medium or pH 6.2 medium + Mel (100 µM) for 24 h and CCK-8 was performed to detect cell viability. N = 3/group. (**B**,**C**) The Western blotting of cytoplasmic and nuclear TFEB and quantitative analysis. (**D**,**E**) Immunofluorescence was performed to detect the distribution of TFEB in SH-SY5Y cells. TFEB signals in the nucleus were quantified. (Scale bar = 20 µm). (**F**,**G**) The Western blotting of P62, LC3, PSD-95, synaptophysin, Caspase-3, and cleaved Caspase-3 and quantitative analysis. (**H**,**I**) PI staining was performed to validate and quantify the cell death ratio (Scale bar = 20 µm). Data are presented as means ± SD. ** *p* < 0.01, *** *p* < 0.001 vs. Con, # *p* < 0.05, ## *p* < 0.01, ### *p* < 0.001 vs. pH 6.2, N = 4/group.

Based on previous studies, 100 µM melatonin was selected to explore its protective effect [28]. Figure 2A showed that SH-SY5Y cells treated with melatonin exhibited higher viability after acid treatment. Nuclear translocation of TFEB was restored following melatonin treatment, as confirmed using Western blotting and immunofluorescence staining (Figure 2B–E). Additionally, melatonin reversed the reduction in autophagy levels in SH-SY5Y cells caused by the acidic environment, as indicated by decreased p62 levels and increased LC3-II/LC3-I levels (Figure 2F,G). Compared with the pH 6.2 group, melatonin restored the levels of PSD-95 and synaptophysin (Figure 2F,G). Furthermore, Western blotting showed reduced levels of cleaved Caspase-3 (Figure 2F,G) and PI staining revealed a decreased proportion of PI-positive cells in the pH 6.2 + Mel group (Figure 2H,I). These findings indicate that melatonin restored TFEB nuclear translocation and autophagy and alleviated neuronal damage caused by acid treatment.

### 2.3. TFEB Nuclear Translocation and Autophagy Were Inhibited in the PT-Stroke Mice

**Figure 3:**
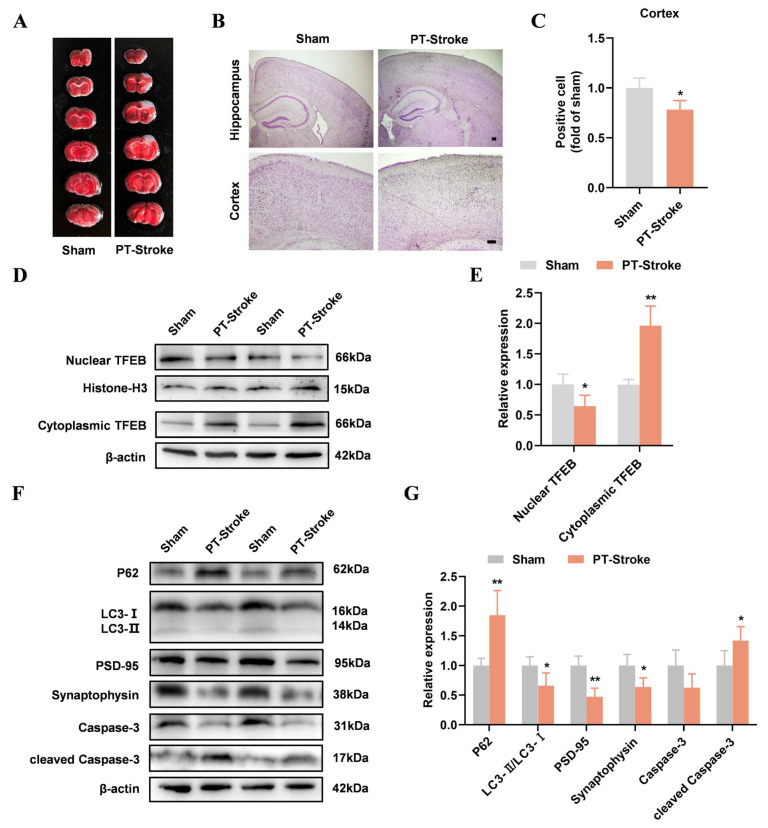
TFEB nuclear translocation and autophagy were inhibited in the PT-stroke mice. (**A**) TTC staining of brain sections (N = 4). (**B**) Nissl staining was performed to measure the number of neurons in the cortex region. (**C**) The Nissl-positive cells in the cortex were quantified using ImageJ. Scale bar = 50 µm (N = 3). (**D**,**E**) The Western blotting of cytoplasmic and nuclear TFEB and quantitative analysis. (**F**,**G**) The Western blotting of P62, LC3, PSD-95, synaptophysin, Caspase-3, and cleaved Caspase-3 and quantitative analysis. Data are presented as means ± SD. * *p* < 0.05, ** *p* < 0.01 vs. Sham, N = 4/group.

In experiment 1, the PT-Stroke group underwent photothrombotic modeling. TTC staining confirmed cerebral ischemia in PT-stroke mice, indicating successful PT-stroke modeling (Figure 3A). There was a decrease in the number of Nissl-positive neurons in the cortex of mice with PT-stroke (Figure 3B,C). Compared with the Sham group, cytoplasmic TFEB levels increased while nuclear TFEB levels decreased in the PT-Stroke group (Figure 3D,E), suggesting the inhibition of TFEB nuclear translocation in mice with PT-stroke. Increased p62 levels and decreased LC3-II/LC3-I levels were observed in the cortices of mice with PT-stroke (Figure 3F,G). Additionally, PSD-95 and synaptophysin levels were reduced, while cleaved Caspase-3 levels were elevated in PT-stroke mice (Figure 3F,G). These results suggest that autophagy inhibition and neuronal damage occurred in mice with PT-stroke.

### 2.4. Melatonin Restored TFEB Nuclear Translocation and Autophagy in Mice with PT-Stroke

**Figure 4:**
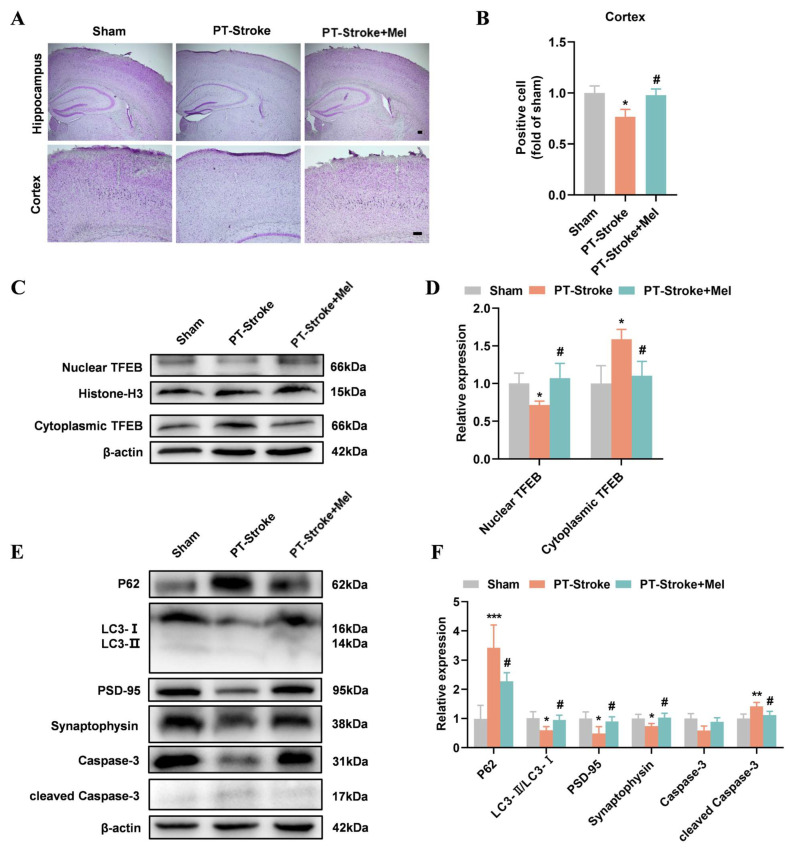
Melatonin restored TFEB nuclear translocation and autophagy in PT-stroke mice. (**A**) Nissl staining was performed to measure the number of neurons in the cortex region. (**B**) The Nissl-positive cells in the cortex were quantified using ImageJ. Scale bar = 50 µm (N = 3). (**C**,**D**) The Western blotting of cytoplasmic and nuclear TFEB and quantitative analysis. (**E**,**F**) The Western blotting of P62, LC3, PSD-95, synaptophysin, Caspase-3, and cleaved Caspase-3 and quantitative analysis. Data are presented as means ± SD. * *p* < 0.05, ** *p* < 0.01, *** *p* < 0.001 vs. Sham, # *p* < 0.05 vs. PT-Stroke, N = 4/group.

In experiment 2, the PT-stroke mice were treated with melatonin for 21 days. Nissl staining showed that mice in the PT-Stroke + Mel group had more positive neurons in the cortex compared with the PT-Stroke group (Figure 4A,B). Western blotting revealed that melatonin promoted TFEB nuclear translocation and autophagy in mice in the PT-Stroke + Mel group, evidenced by decreased cytoplasmic TFEB and p62 levels, as well as increased nuclear TFEB and LC3-II/LC3-I levels (Figure 4C–F). Moreover, PSD-95 and synaptophysin levels were restored, while cleaved Caspase-3 levels were reduced after melatonin treatment (Figure 4E,F). These findings indicate that melatonin restores autophagy and alleviates nerve damage in mice with PT-stroke.

### 2.5. TFEB Knockdown Down-Regulated Autophagy and Blocked the Protective Effect of Melatonin on Neurons

**Figure 5:**
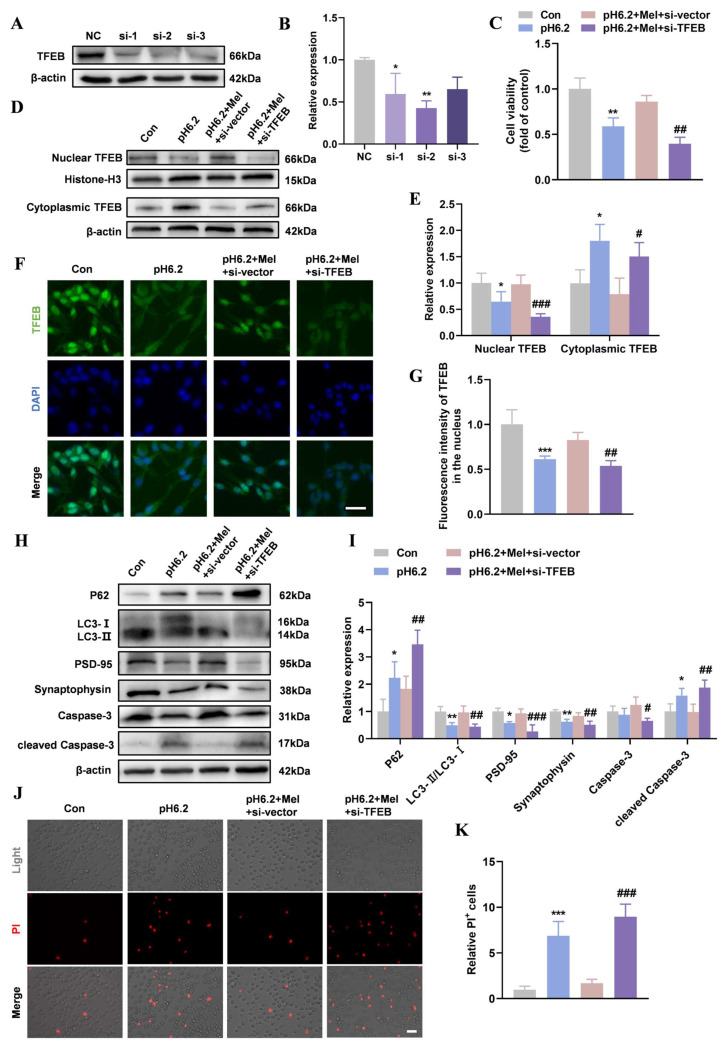
TFEB knockdown down-regulated autophagy and blocked the protective effect of melatonin on neurons. (**A**,**B**) SH-SY5Y cells were infected with three si-TFEBs (si-1, si-2, and si-3). Interference efficiency was determined using Western blotting. (**C**) SH-SY5Y cells were treated with pH 6.2 medium or pH 6.2 medium + Mel (100 µM) + si-vector or pH 6.2 medium + Mel (100 µM) + si-TFEB for 48 h and CCK-8 was performed to detect cell viability. N = 3/group. (**D**,**E**) The Western blotting of cytoplasmic and nuclear TFEB and quantitative analysis. (**F**,**G**) Immunofluorescence was used to detect the distribution of TFEB in SH-SY5Y cells. TFEB signals in the nucleus were quantified. (Scale bar = 20 µm). (**H**,**I**) The Western blotting of P62, LC3, PSD-95, synaptophysin, Caspase-3, and cleaved Caspase-3 and quantitative analysis. (**J**,**K**) PI staining was performed to validate and quantify the cell death ratio (Scale bar = 20 µm). Data are presented as means ± SD. * *p* < 0.05, ** *p* < 0.01, *** *p* < 0.001 vs. Con or NC, # *p* < 0.05, ## *p* < 0.01, ### *p* < 0.001 vs. pH 6.2 + Mel + si-vector, N = 4/group.

Western blotting confirmed the specific downregulation of TFEB expression in SH-SY5Y cells transduced with siRNAs (si-1, si-2, and si-3) targeting TFEB (Figure 5A,B). TFEB levels were significantly reduced in the si-2 group, which was selected for subsequent experiments. Figure 5C showed that SH-SY5Y cell viability decreased in the pH 6.2 + Mel + si-TFEB group. Western blotting and immunofluorescence revealed a significant decrease in TFEB nuclear translocation (Figure 5D–G). Synaptic damage and increased apoptosis were evident in the pH 6.2 + Mel + si-TFEB group, as indicated by reduced PSD-95 and synaptophysin levels, along with increased cleaved Caspase-3 levels and PI-positive cells (Figure 5H–K). These findings suggest that TFEB knockdown down-regulates autophagy and blocks the protective effects of melatonin on neurons.

### 2.6. TFEB Overexpression Promoted Autophagy and Protected Neurons in an Acidic Environment

**Figure 6:**
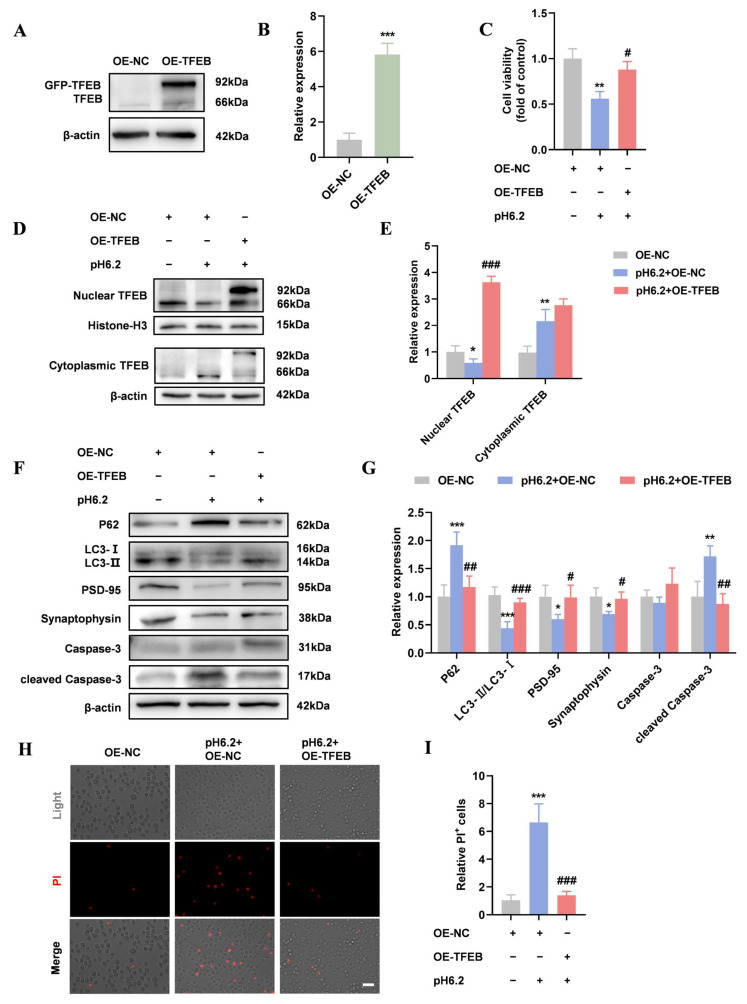
Overexpression of TFEB promoted autophagy and protected neurons in acidic environments. (**A**,**B**) For overexpression of TFEB (OE-TFEB), SH-SY5Y cells were infected with GFP-TFEB. For negative control (OE-NC), SH-SY5Y cells were infected with GFP-vector. Interference efficiency was determined using Western blotting. (**C**) SH-SY5Y cells were treated with pH 6.2 medium + GFP vector or pH 6.2 medium + GFP-TFEB for 48 h and CCK-8 was performed to detect cell viability. N = 3/group. (**D**,**E**) The Western blotting of cytoplasmic and nuclear TFEB and quantitative analysis. (**F**,**G**) The Western blotting of P62, LC3, PSD-95, synaptophysin, Caspase-3, and cleaved Caspase-3 and quantitative analysis. (**H**,**I**) PI staining was performed to validate and quantify the cell death ratio (Scale bar = 20 µm). Data are presented as means ± SD. * *p* < 0.05, ** *p* < 0.01, *** *p* < 0.001 vs. OE-NC, # *p* < 0.05, ## *p* < 0.01, ### *p* < 0.001 vs. pH 6.2 + OE-NC, N = 4/group.

SH-SY5Y cells were transfected with GFP-TFEB or GFP-vector. The Western blotting results showed a significant increase in TFEB levels in GFP-TFEB-transfected SH-SY5Y cells (Figure 6A,B). Overexpression of TFEB slightly restored cell viability and TFEB nuclear translocation in SH-SY5Y cells under acidic conditions (Figure 6C–E). Compared with the pH6.2 + OE-NC group, TFEB upregulation enhanced autophagy, as indicated by reduced p62 and elevated LC3-II/LC3-I levels (Figure 6F,G). Additionally, increased PSD-95 and synaptophysin levels, along with reduced cleaved Caspase-3 and PI-positive cells, were observed in the pH6.2 + GFP-TFEB group (Figure 6F–I). These findings suggest that TFEB overexpression promotes nuclear translocation and autophagy while mitigating synaptic damage and apoptosis.

## 3. Discussion

Acidosis is a critical pathophysiological condition that arises following tissue ischemia and hypoxia. In this study, SH-SY5Y cells were exposed to an acidic environment (pH 6.2, 24 h) to simulate acidosis and the PT method was used to establish an acidosis animal model of cerebral ischemic stroke. This study demonstrated that acidosis leads to increased cytoplasmic TFEB levels, decreased nuclear TFEB levels, and impaired autophagy. Melatonin promotes autophagy by enhancing TFEB nuclear translocation, protecting synapses, and reducing apoptosis, thereby exerting a protective effect on neurons under acidic conditions. Conversely, TFEB downregulation blocked the neuroprotective effects of melatonin.

Previous studies have shown that autophagy dysfunction occurs in neurons in the brains of stroke-affected mice and that acidic environments disrupt lysosomal signaling pathways [28,32,33]. In our study, autophagy dysfunction was observed in both animal and cellular models of acidosis. Specifically, levels of the autophagy marker LC3-II/LC3-I were reduced, while the levels of the autophagy substrate degradation marker P62 were elevated, indicating autophagy impairment in both in vivo and in vitro models of acidic environments. Notably, prior studies have suggested that autophagy may be transiently activated during the early stages of ischemia. However, despite initial upregulation, impaired lysosomal function often results in compromised autophagy at later stages [33]. Autophagy is a dynamic, complex process and insufficient and excessive autophagy can harm neuronal health [34,35]. Our previous study showed that excessive autophagy activity in hippocampal neurons following stroke was linked to cognitive impairment [29]. Thus, the balance of autophagy activity is crucial, as its activation or inhibition can be a precursor to long-term dysfunction.

Dysfunctional autophagy has been reported to disturb synaptic proteins, leading to synaptic dysfunction [36]. Global autophagy dysfunction in *Atg7*iKO mice disrupts synaptic homeostasis and impairs cognitive function, resulting in early changes associated with cognitive impairments such as AD [37]. Impaired autophagy, leading to tau hyperphosphorylation and synaptic dysfunction, has also been observed in mice fed a high-fat diet and in N2a cells treated with PA medium [38]. Our study showed that synapse-related protein PSD-95 and synaptophysin levels decreased under acidic conditions, indicating synaptic damage.

Autophagy is a complex process regulated by various signaling pathways and factors, with TFEB being a key regulator [39]. In several neurodegenerative disease models, TFEB plays a key role in enhancing autophagy, thereby alleviating disease progression [40,41,42,43,44]. Small-molecule TFEB activators or TFEB overexpression can enhance autophagy and attenuate APP/Aβ and MAPT/Tau pathology in animal models [45,46,47]. Similarly, TFEB-dependent autophagy enhancers, such as Torin1 and the curcumin analog C1, reverse cell death in a PD model [48]. Moreover, in a permanent MCAO (pMCAO) model, nuclear TFEB levels and lysosome activity gradually decreased, accumulating autophagosomes and autophagy substrates, which worsened ischemic injury [43]. Neuron-specific TFEB overexpression significantly enhanced autophagy and reduced ischemic injury. However, TFEB knockdown significantly reversed autophagy activation and aggravated neurological deficits and ischemic outcomes in the pMCAO model [49]. These findings highlight the critical role of TFEB in regulating autophagy. Similarly, our study demonstrated that acidic environments inhibited TFEB nuclear translocation, leading to impaired autophagy. Overexpression of TFEB restored autophagy and reduced apoptosis in acidic conditions.

Melatonin is widely used in clinical practice and basic scientific studies and has a high safety profile. Importantly, melatonin is an ideal neuroprotective agent. No rebound or inhibition of endogenous melatonin production has been observed after discontinuation [50,51,52]. The therapeutic potential of melatonin as an inducer of autophagy via TFEB activation has been proposed in models of nervous system diseases. Melatonin prevents the nerve cell death caused by cadmium exposure, by enhancing TFEB expression and nuclear translocation [53]. Additionally, melatonin promotes mitochondrial autophagy by inducing TFEB nuclear translocation, thereby protecting SH-SY5Y cells and APP/PS1 mice [54]. Melatonin reverses acidosis-induced neuronal death, reduced synaptic protein levels, tau hyperphosphorylation, and kinase/phosphatase imbalance in acidic environments [28]. Our study reveals that melatonin promotes the nuclear translocation of TFEB, thereby enhancing autophagy and protecting neurons under acidic conditions. Contrary to our findings in the cortex of mice with PT-stroke, melatonin can inhibit autophagy overactivation in the hippocampus of stroke mice, thereby improving cognitive impairment after stroke [29]. Our further studies will focus on exploring the distinct mechanisms of injury in different brain regions following ischemia to develop targeted treatments for different post-stroke injuries.

There may be some limitations in this study that could be addressed in future research. First, autophagy flux assays, such as those using lysosomal inhibitors, were not performed. Our study focused on the impact of TFEB on autophagy. The results confirmed that autophagy in neurons in an acidic environment was inhibited, and that promoting the nuclear translocation of TFEB could restore autophagy. Conducting related studies with autophagy flux assays would make our research more comprehensive, enabling us to confirm autophagic degradation efficiency. This would provide a deeper understanding of how TFEB affects autophagy and identify which specific processes of autophagy are influenced. Second, despite its lack of serious toxicity over a wide dose range, further extensive and detailed studies should be performed to achieve the most effective and safest method of melatonin administration regarding dose and duration of use.

## 4. Materials and Methods

### 4.1. Animals

Male C57/BL6J mice at 8 weeks were purchased from Hunan Slake Jingda Laboratory Animal Co., Ltd. (Changsha, China). Animals were housed in cages with free access to water and food at 22 ± 2 °C under a 12 h light–dark cycle. All procedures and treatments were approved by the Animal Care and Use Committee of Hunan Normal University.

### 4.2. Photothrombotic (PT) Infarction Models and Treatment

PT models were established as described in our previous studies [29]. Briefly, anesthetized mice were injected with Rose Bengal (10 mg/mL in 0.9% saline; Sigma-Aldrich, St. Louis, MO, USA). The head hair was shaved, and the mice were placed in a brain stereotaxic apparatus (David Kopf Instruments, Tujunga, CA, USA). The skulls were exposed and irradiated with a cold light source at a fixed point for 20 min, located 2 mm lateral to the right of the bregma.

For experiment 1, mice were randomized into Sham and PT-Stroke groups (n = 11/group). The PT-Stroke group underwent photothrombotic modeling, while the Sham group received an intraperitoneal injection of Rose Bengal without illumination.

For experiment 2, mice were randomized into Sham, PT-Stroke, and PT-Stroke + Mel groups (n = 7/group). Melatonin (Mel, 20 mg/kg; Sigma-Aldrich, St. Louis, MO, USA) was dissolved in 0.9% saline. After 24 h of the PT modeling, mice in the PT-Stroke + Mel group were intraperitoneally injected with melatonin for 21 days. Mice in the Sham and PT-Stroke groups were injected with equal saline for 21 days.

### 4.3. 2,3,5-Triphenyl-Tetrazolium Chloride (TTC) Staining

Frozen whole-brain tissue was sectioned into six evenly thick coronal slices (approximately 2 mm each). The slices were placed in a glass beaker containing 2% TTC solution (MeilunBio, Dalian, China) and incubated in a 37 °C water bath for 10 min. The slices were flipped once during staining, and the process was conducted shielded from light.

### 4.4. Nissl Staining

Following gradient dehydration with 10% and 30% sucrose solutions, mouse brains were sectioned into 30 µm slices using a frozen sectioning machine (Leica, Wetzlar, Germany). According to the manufacturer’s instructions, hippocampal and cortical sections were stained using a Nissl staining kit (Solarbio, Beijing, China). The images were statistically analyzed using the ImageJ 1.53 software.

### 4.5. Cell Culture

SH-SY5Y cells were grown in Dulbecco’s Modified Eagle Medium (DMEM; Gibco, NY, USA) supplemented with 10% fetal bovine serum (FBS; Cell-Box, Hong Kong, China) at 37 °C in a 5% CO_2_ atmosphere. The culture medium was replaced every 24 h. When the cell density reached around 80%, a cell passage was performed. Cells were routinely verified to be free of mycoplasma contamination.

Media at varying pH values (6.5, 6.2, and 6.0) were prepared by titration with HCl/NaOH in DMEM. SH-SY5Y cells were treated with acid medium for 24 h.

### 4.6. Cell Counting Kit-8 (CCK-8) Assay

SH-SY5Y cells were seeded in 96-well plates at a density of 5 × 10^3^ cells/well. Following 24 h treatment, CCK-8 reagent (APExBIO, Houston, TX, USA) was added to each well. After a 4 h incubation, the optical density was at 450 nm using a microplate reader (Heales, Shenzhen, China).

### 4.7. Transfection

SH-SY5Y cells were transiently transfected with GFP-TFEB plasmids (Sino Biological, Beijing, China) for 48 h or siRNA (HANBIO, Shanghai, China) targeting the autophagy gene TFEB for 72 h, using Lipo8000™transfection reagent (Beyotime, Shanghai, China), following the manufacturer’s protocol.

### 4.8. Propidium Iodide (PI) Staining

SH-SY5Y cells were washed with phosphate-buffered saline, counted, and 1 × 10^5^ cells were used for the subsequent steps. Following the manufacturer’s instructions, apoptosis was visualized using a PI kit (UElandy, Suzhou, China) under a fluorescence microscope.

### 4.9. Immunofluorescence Staining

SH-SY5Y cells were fixed with 4% paraformaldehyde (Biosharp, Hefei, China) for 15 min and incubated with 0.2% Triton X-100 (Yeasen, Shanghai, China) for 10 min at room temperature. After blocking with 5% bovine serum albumin for an hour, cells were incubated overnight at 4 °C with TFEB antibody (Beyotime, Shanghai, China; 1:50). The binding of the primary antibodies was visualized using iFluor 488-conjugated goat anti-rabbit IgG antibody (HUABIO, Hangzhou, China; 1:800). Fluorescence images were captured using a fluorescence microscope (Thermo Fisher Scientific, Waltham, MA, USA).

### 4.10. Western Blotting

The cells and cortical tissues were lysed in a radio immunoprecipitating buffer (NCM Biotech, Suzhou, China) containing protease phenylmethylsulfonyl fluoride. The lysates were denatured in a loading buffer and separated using 10%, 12.5%, or 15% of SDS-PAGE gels. The proteins were transferred onto polyvinylidene fluoride membranes and blocked with 5% non-fat milk. The membranes were incubated overnight with primary antibodies: anti-TFEB (Beyotime, Shanghai, China; 1:1000), anti-Histone-H3 (zenbio, Chengdu, China; 1:5000), anti-β-actin (ABclonal, Wuhan, China; 1:40,000), anti-P62 (HUABIO, Hangzhou, China; 1:1000), anti-LC3 (Proteintech, Wuhan, China; 1:1000), anti-PSD-95 (HUABIO, Hangzhou, China; 1:1000), anti-synaptophysin (Beyotime, Shanghai, China; 1:1000), anti-Caspase-3 (ABclonal, Wuhan, China; 1:1000), and anti-cleaved Caspase-3 (Abmart, Shanghai, China; 1:1000). The membranes were then incubated with secondary anti-rabbit or anti-mouse antibodies (APExBIO, Houston, TX, USA; 1:10,000) at room temperature for approximately 1.5 h. Immunoblots were visualized using the ECL Chemiluminescent Substrate Detection Kit (APExBIO, Houston, TX, USA) and detected with an imaging system (Tanon-5200, Shanghai, China). Furthermore, grayscale analysis was conducted using ImageJ 1.53 software.

### 4.11. TFEB Content Analysis in Nucleus and Cytoplasm

The cells were washed with PBS and the cortical tissue was made into a cell suspension using a homogenizer. Following the manufacturer’s protocol, the nuclear and cytoplasmic extract was isolated using NE-PER Nuclear and Cytoplasmic Extraction Reagents (Solarbio, Beijing, China). Western blotting was then conducted, as previously described.

### 4.12. Statistical Analysis

The data were presented as mean ± standard deviation (SD). Statistical analyses were performed using the unpaired Student’s *t*-test or one-way analysis of variance with GraphPad Prism 5. A *p*-value of <0.05 was considered statistically significant.

## 5. Conclusions

In conclusion, these results confirm that melatonin promotes autophagy and protects neurons from damage in acidic environments. These findings highlight the significance of targeting TFEB as a key regulator of autophagy dysfunction and ischemic injury caused by acidosis. TFEB emerges as a promising target for neuroprotective therapy in cerebral ischemia. The protective effect of melatonin on cerebral ischemia may involve several mechanisms. This study provides valuable insights into the acidosis pathophysiology by elucidating the mechanism through which melatonin regulates autophagy. It offers a potential therapeutic approach for neurological diseases associated with acidosis.

## Data Availability

The raw data supporting the conclusions of this article will be made available by the corresponding authors without undue reservation.
